# Supplementary material for: A global ecological signal of extinction risk in marine ray-finned fishes (class Actinopterygii)
Source: Camb Prism Extinct. 2023 Nov 14;1:e25. doi: 10.1017/ext.2023.23 (PMC11895746; doi:10.1017/ext.2023.23)
Supplement: Bak et al. supplementary material [file S2755095823000232sup001.docx]

Supporting Information

Supplemental Table 1. AIC model selection, with median and 95% confidence intervals for ΔAIC across all 100 phylogenetic trees; minimum AIC is 703.36. W_i_ is the AIC weights for each model and shows the proportion of model support.

| Variables | Median ΔAIC | Δ Lower  95% CI | Δ Upper 95% CI | W_i_ |
| --- | --- | --- | --- | --- |
| Total Length, Euryhaline Status | 0.00 | 0.00 | 1.69 | 0.68 |
| Euryhaline Status | 1.99 | 0.00 | 6.14 | 0.25 |
| Total Length, Minimum Population Doubling Time, Euryhaline Status | 5.43 | 2.15 | 17.09 | 0.05 |
| Total Length, Minimum Population Doubling Time, Tiering, Euryhaline Status | 7.12 | 3.29 | 26.27 | 0.02 |
| Total Length | 14.47 | 7.52 | 23.54 | 0.00 |
| Trophic Level | 19.17 | 9.84 | 33.53 | 0.00 |
| Tiering | 22.86 | 7.85 | 43.24 | 0.00 |
| Minimum Population Doubling Time | 27.62 | 12.59 | 36.36 | 0.00 |
| Total Length, Trophic Level, Minimum Population Doubling Time, Tiering, Euryhaline Status | 28.24 | 6.31 | 43.21 | 0.00 |
| (Null) | 33.15 | 22.04 | 45.23 | 0.00 |

Supplemental Table 2. PhyloGLM results table; median model estimate, averaged standard error (Avg SE), and lower and upper 95% confidence intervals (CI) across all 100 phylogenetic trees are reported for PhyloGLMs.

|  | PhyloGLM | | | |
| --- | --- | --- | --- | --- |
| Variable  (Level of Variable) | Median Estimate | Avg SE | Lower 95% CI | Upper 95% CI |
| Total Length (log)mm | 0.67 | 0.25 | 0.51 | 1.16 |
| Euryhaline Status (Marine and Brackish) | -0.35 | 0.26 | -0.86 | 0.16 |
| Euryhaline Status (Marine, Brackish, and Freshwater) | 0.94 | 0.34 | 0.26 | 1.61 |

Supplemental Table 3. Critically endangered species, sorted by total length, from largest to smallest, with euryhaline status.

| Scientific Name | Scientific Family Name | Common Family Name | Total Length (mm) | Euryhaline Status |
| --- | --- | --- | --- | --- |
| *Huso dauricus* | Acipenseridae | Sturgeons | 5600 | Marine Brackish and Freshwater |
| *Acipenser sturio* | Acipenseridae | Sturgeons | 5000 | Marine Brackish and Freshwater |
| *Huso huso* | Acipenseridae | Sturgeons | 5000 | Marine Brackish and Freshwater |
| *Acipenser schrenckii* | Acipenseridae | Sturgeons | 3000 | Marine Brackish and Freshwater |
| *Stereolepis gigas* | Polyprionidae | Wreckfishes | 2500 | Marine Only |
| *Acipenser gueldenstaedtii* | Acipenseridae | Sturgeons | 2350 | Marine Brackish and Freshwater |
| *Acipenser persicus* | Acipenseridae | Sturgeons | 2300 | Marine Brackish and Freshwater |
| *Acipenser stellatus* | Acipenseridae | Sturgeons | 2200 | Marine Brackish and Freshwater |
| *Acipenser naccarii* | Acipenseridae | Sturgeons | 2000 | Marine Brackish and Freshwater |
| *Acipenser nudiventris* | Acipenseridae | Sturgeons | 2000 | Marine Brackish and Freshwater |
| *Bahaba taipingensis* | Sciaenidae | Drums and croakers | 2000 | Marine and Brackish |
| *Acipenser mikadoi* | Acipenseridae | Sturgeons | 1500 | Marine Brackish and Freshwater |
| *Anguilla anguilla* | Anguillidae | Eels | 1330 | Marine Brackish and Freshwater |
| *Acipenser sinensis* | Acipenseridae | Sturgeons | 1300 | Marine Brackish and Freshwater |
| *Epinephelus striatus* | Serranidae | Groupers and Sea Basses | 1220 | Marine Only |
| *Polysteganus undulosus* | Sparidae | Sea Breams | 1200 | Marine Only |
| *Coryphaenoides rupestris* | Macrouridae | Grenadiers | 1100 | Marine Only |
| *Larimichthys crocea* | Sciaenidae | Drums and croakers | 800 | Marine and Brackish |
| *Chrysoblephus cristiceps* | Sparidae | Sea Breams | 750 | Marine Only |
| *Pseudoscaphirhynchus kaufmanni* | Acipenseridae | Sturgeons | 750 | Marine Brackish and Freshwater |
| *Pseudoscaphirhynchus fedtschenkoi* | Acipenseridae | Sturgeons | 650 | Marine Brackish and Freshwater |
| *Coregonus huntsmani* | Salmonidae | Salmon | 400 | Marine Brackish and Freshwater |
| *Pseudoscaphirhynchus hermanni* | Acipenseridae | Sturgeons | 275 | Marine Brackish and Freshwater |
| *Brachionichthys hirsutus* | Brachionichthyidae | Handfishes | 150 | Marine Only |
| *Syngnathus watermeyeri* | Syngnathidae | Seahorses | 130 | Marine and Brackish |
| *Hypomesus transpacificus* | Osmeridae | Smelts | 120 | Marine Brackish and Freshwater |
| *Allotoca diazi* | Goodeidae | Splitfins | 100 | Marine Brackish and Freshwater |
| *Knipowitschia cameliae* | Gobiidae | Gobies | 31 | Marine and Brackish |
| *Aphanius almiriensis* | Cyprinodontidae | Pupfishes | NA | Marine and Brackish |
| *Acanthobrama centisquama* | Cyprinidae | Minnows | NA | Marine Brackish and Freshwater |
| *Alburnus nasreddini* | Cyprinidae | Minnows | NA | Marine Brackish and Freshwater |
| *Azurina eupalama* | Pomacentridae | Damselfishes | NA | Marine Only |
| *Gobulus birdsongi* | Gobiidae | Gobies | NA | Marine Only |
| *Limia rivasi* | Poeciliidae | Guppies and mollies | NA | Marine Brackish and Freshwater |
| *Lucifuga simile* | Bythitidae | Brotulas | NA | Marine Brackish and Freshwater |
| *Luciogobius albus* | Gobiidae | Gobies | NA | Marine Brackish and Freshwater |
| *Neostethus ctenophorus* | Phallostethidae | Priapiums | NA | Marine Brackish and Freshwater |
| *Neostethus robertsi* | Phallostethidae | Priapiums | NA | Marine Brackish and Freshwater |
| *Paraclinus walkeri* | Labrisomidae | Blennies | NA | Marine Only |
| *Poropuntius chonglingchungi* | Cyprinidae | Minnows | NA | Marine Brackish and Freshwater |
| *Probarbus jullieni* | Cyprinidae | Minnows | NA | Marine Brackish and Freshwater |
| *Proterorhinus tataricus* | Gobiidae | Gobies | NA | Marine Brackish and Freshwater |
| *Scaphirhynchus suttkusi* | Acipenseridae | Sturgeons | NA | Marine Brackish and Freshwater |
| *Sciaena callaensis* | Sciaenidae | Croakers | NA | Marine Only |
| *Takifugu chinensis* | Tetraodontidae | Pufferfishes | NA | Marine Only |

Supplemental Table 4. Dunn Test for total length pair comparisons among International Union for Conservation of Nature (IUCN) categories. CR, critically endangered; EN, endangered, VU, vulnerable.

| Comparison | Z | P Value |
| --- | --- | --- |
| CR - EN | 3.64 | < 0.01 |
| CR - Non-Threatened | 6.05 | < 0.001 |
| EN - Non-Threatened | 1.54 | 0.74 |
| CR - VU | 4.82 | < 0.001 |
| EN - VU | 0.59 | 1.0 |
| Non-Threatened - VU | -1.52 | 0.77 |

Table 5. Standardized residuals from chi-square test for International Union for Conservation of Nature (IUCN) categories. CR, critically endangered; EN, endangered, VU, vulnerable.

| Euryhaline Status | Non-Threatened | VU | EN | CR |
| --- | --- | --- | --- | --- |
| Marine and Brackish | -1.82 | 0.58 | 2.40 | 0.34 |
| Marine Brackish and Freshwater | -11.61 | 3.46 | 8.64 | 12.44 |
| Marine Only | 10.06 | -3.03 | -8.27 | -9.58 |

Supplemental Table 6. PhyloGLM best model association with threat types. SE is Standard Error, L95 is Lower 95% confidence interval and U95 is Upper 95% confidence interval

| Variable (Level of Variable) | Median Coefficient | SE | L95 | U95 |
| --- | --- | --- | --- | --- |
| **Harvesting** | | | | |
| Total Length (log)mm | 2.32 | 0.23 | 1.86 | 2.78 |
| Euryhaline Status (Marine and Brackish) | 0.00 | 0.13 | -0.26 | 0.25 |
| Euryhaline Status (Marine, Brackish, and Freshwater) | -0.88 | 0.22 | -1.30 | -0.45 |
| **Development** | | | | |
| Total Length (log)mm | -1.16 | 0.45 | -2.04 | -0.27 |
| Euryhaline Status (Marine and Brackish) | 0.85 | 0.31 | 0.24 | 1.47 |
| Euryhaline Status (Marine, Brackish, and Freshwater) | 1.17 | 0.33 | 0.51 | 1.82 |
| **Pollution** | | | | |
| Total Length (log)mm | 0.12 | 0.75 | -1.36 | 1.59 |
| Euryhaline Status (Marine and Brackish) | 0.33 | 0.31 | -0.27 | 0.94 |
| Euryhaline Status (Marine, Brackish, and Freshwater) | 0.15 | 0.32 | -0.48 | 0.78 |
| **Climate Change** | | | | |
| Total Length (log)mm | -0.16 | 0.04 | -0.24 | -0.07 |
| Euryhaline Status (Marine and Brackish) | 1.21 | 0.06 | 1.09 | 1.33 |
| Euryhaline Status (Marine, Brackish, and Freshwater) | 2.02 | 0.07 | 1.88 | 2.17 |
